# Efficacy of Sublingual Misoprostol Versus Intravenous Tranexamic Acid in Reducing Blood Loss Among Full-Term Pregnant Women Undergoing Primary Lower Segment Caesarean Section: A Randomized Controlled Trial

**DOI:** 10.7759/cureus.114326

**Published:** 2026-08-11

**Authors:** Nikhitha Basavaraj, Ashwini B., Kotur Pushpa

**Affiliations:** 1 Obstetrics and Gynaecology, Adichunchanagiri Institute of Medical Sciences, Adichunchanagiri University, B. G. Nagara, IND; 2 Obstetrics and Gynaecology, Aarupadai Veedu Medical College and Hospital, Puducherry, IND

**Keywords:** caesarean section, misoprostol, postpartum haemorrhage, tranexamic acid, uterotonic agents

## Abstract

Introduction: Postpartum hemorrhage remains a major cause of maternal morbidity and mortality, with caesarean section being associated with increased intraoperative blood loss. Pharmacological agents such as misoprostol and tranexamic acid have been used prophylactically to reduce blood loss during lower segment caesarean section (LSCS); however, evidence directly comparing their efficacy is limited. This study aimed to compare the efficacy and safety of sublingual misoprostol and intravenous tranexamic acid in reducing intraoperative blood loss during primary LSCS.

Materials and methods: This hospital-based, open-label, randomized controlled trial included 140 women with singleton term pregnancies undergoing primary LSCS. Participants were randomized equally to receive either 600 µg sublingual misoprostol or 1 g intravenous tranexamic acid immediately before skin incision. The primary outcome was intraoperative blood loss measured using a standardized gravimetric method. Secondary outcomes included postoperative fall in haemoglobin, requirement for additional uterotonic agents or blood transfusion, and adverse drug effects.

Results: Baseline demographic and obstetric characteristics were comparable between the two groups. Mean intraoperative blood loss was significantly lower in the misoprostol group than in the tranexamic acid group (316.0 ± 158.0 mL vs. 518.5 ± 233.1 mL; p < 0.001). Women receiving misoprostol also had a significantly smaller postoperative fall in haemoglobin and required fewer additional uterotonic agents (2 (2.9%) vs. 11 (15.7%); p = 0.009) and blood transfusions (1 (1.4%) vs. 9 (12.9%); p = 0.016). Fever and shivering were more common with misoprostol, whereas hypotension occurred more frequently in the tranexamic acid group (all p < 0.05).

Conclusion: Sublingual 600 µg misoprostol was more effective than 1 g intravenous tranexamic acid in reducing intraoperative blood loss during primary LSCS. It was associated with a smaller postoperative decline in haemoglobin and a reduced need for additional uterotonic agents and blood transfusion, while maintaining an acceptable safety profile. These findings support the use of sublingual misoprostol as an effective and economical option for preventing excessive blood loss during primary LSCS.

## Introduction

Postpartum haemorrhage (PPH) remains one of the leading causes of maternal morbidity and mortality worldwide and is recognized as a major obstetric emergency [1]. Despite advances in obstetric care, PPH continues to account for a substantial proportion of maternal deaths, particularly in low- and middle-income countries, where nearly 99% of PPH-related maternal deaths occur [2]. In India, obstetric haemorrhage contributes to approximately 19.9% of all maternal deaths, underscoring the need for effective preventive strategies during both vaginal and operative deliveries [3]. Caesarean section is associated with greater blood loss than vaginal delivery, and the increasing global rate of caesarean deliveries has further emphasized the importance of effective strategies for preventing excessive perioperative blood loss [4,5].

Uterine atony is the most common cause of PPH, accounting for nearly half of all cases [6]. Consequently, prophylactic administration of uterotonic agents forms the cornerstone of PPH prevention [7]. The World Health Organization recommends active management of the third stage of labour, including the routine administration of oxytocin as the first-line uterotonic agent [8]. However, clinically significant blood loss may still occur despite prophylactic uterotonic administration, highlighting the need for additional pharmacological strategies to reduce perioperative blood loss and minimize the requirement for blood transfusion or surgical intervention [9].

Several pharmacological agents have been evaluated as adjuncts to conventional uterotonics for reducing blood loss during caesarean section [10]. Tranexamic acid (TXA), a synthetic antifibrinolytic agent, inhibits fibrinolysis and has been shown to reduce perioperative blood loss across various surgical specialties, including obstetrics [11,12]. Misoprostol, a prostaglandin E1 analogue with potent uterotonic properties, offers advantages such as ease of administration, rapid absorption through the sublingual route, low cost, and stability at room temperature, making it particularly useful in resource-limited settings [13,14]. Both TXA and misoprostol have been investigated for reducing blood loss during caesarean delivery; however, their mechanisms of action and adverse-effect profiles differ, and evidence directly comparing their relative efficacy and safety remains limited.

Therefore, the present randomized controlled trial was undertaken to compare the efficacy and safety of 600 µg sublingual misoprostol and 1 g intravenous TXA in women with singleton term pregnancies undergoing primary lower segment caesarean section (LSCS). The primary objective was to compare intraoperative blood loss between the two treatment groups. Secondary objectives were to compare postoperative decline in haemoglobin concentration, the requirement for additional uterotonic agents or blood transfusion, and the incidence of adverse effects associated with the study interventions.

## Materials and methods

This hospital-based randomized controlled trial was conducted in the Department of Obstetrics and Gynaecology, Aarupadai Veedu Medical College and Hospital, Puducherry, India, from July 2021 to December 2022. The study was approved by the Institutional Ethics Committee, Aarupadai Veedu Medical College and Hospital (Approval No. AVMC/IRC/2020/150), and written informed consent was obtained from all participants before enrolment. The trial was prospectively registered with the Clinical Trials Registry-India (CTRI No. CTRI/2021/03/032275).

The sample size was calculated using the formula for comparison of two independent means based on the findings of Karpagam and Bakiavathy, who reported a mean intraoperative blood loss of 190.21 ± 86.64 mL in the misoprostol group and 272.31 ± 102.17 mL in the TXA group [15]. The required sample size was estimated using the formula: $n=\frac{2(Z_{\alpha/2}+Z_{\beta})^2\sigma^2}{(\mu_1-\mu_2)^2}$, assuming a 95% confidence level (α = 0.05) and 99% study power, which yielded a minimum sample size of 50 participants per group. As a greater number of eligible participants were available during the study period, 70 women were enrolled in each group, resulting in a total sample size of 140 participants.

The inclusion criteria were a singleton term pregnancy (≥37 weeks), women undergoing primary LSCS, age ≥18 years, and willingness to provide written informed consent. The exclusion criteria were previous caesarean section, induced or augmented labour, obesity, chorioamnionitis, antepartum haemorrhage, preterm pregnancy, multifetal gestation, polyhydramnios, and uterine over-distension. A total of 140 women with singleton term pregnancies undergoing primary LSCS were enrolled and randomized equally into two groups (70 participants in each group).

Baseline demographic and clinical characteristics, including age, height, weight, body mass index (BMI), obstetric and menstrual history, and findings of general and obstetric examinations, were recorded. All participants underwent routine preoperative investigations and ultrasonography to confirm gestational age and fetal well-being.

Participants were randomized using sequentially numbered, sealed, opaque envelopes, ensuring allocation concealment until enrolment. Owing to the different routes of administration of the study drugs (sublingual misoprostol and intravenous TXA), blinding of the participants and treating clinicians was not feasible; therefore, the study was conducted as an open-label randomized controlled trial. Women allocated to the misoprostol group received 600 µg sublingual misoprostol immediately before skin incision, whereas those allocated to the TXA group received 1 g intravenous TXA diluted in 100 mL normal saline immediately before skin incision. Following delivery of the baby, all participants received 10 IU oxytocin in 500 mL of Ringer's lactate intravenously as standard uterotonic prophylaxis. In cases of excessive intraoperative blood loss or clinically diagnosed uterine atony, additional uterotonic agents and blood transfusion were administered according to a predefined rescue protocol consisting of 800 µg rectal misoprostol, followed sequentially by 20-40 IU oxytocin infusion, 0.25 mg intravenous methylergometrine, and 0.25 mg intramuscular carboprost (15-methyl prostaglandin F2α), depending on the persistence of bleeding [16].

Intraoperative blood loss was estimated using a standardized gravimetric method, in which all surgical drapes, mops, gauze pieces, and pads were weighed before and after surgery, and the difference in weight was converted to blood volume assuming that 1 g weight difference was equivalent to 1 mL of blood loss [17,18]. The calculated blood loss was added to the volume collected in the suction bottle and the volume of blood clots removed from the vagina. To avoid contamination with amniotic fluid, the suction bottle used before delivery of the baby was replaced immediately after delivery, and only the blood collected thereafter was included in the final estimation. Haemoglobin concentration was measured preoperatively and 72 hours after surgery, and the fall in haemoglobin was calculated as the difference between the two measurements.

The primary outcome measure was the total intraoperative blood loss during LSCS. Secondary outcome measures included postoperative decline in haemoglobin concentration, the requirement for additional uterotonic agents or blood transfusion, and adverse effects associated with the study interventions.

The collected data were entered into Microsoft Excel (Microsoft Corp., Redmond, WA, USA) and analysed using IBM SPSS Statistics for Windows, Version 21 (Released 2012; IBM Corp., Armonk, New York, United States). Continuous variables were expressed as mean ± standard deviation (SD) and compared using the independent Student's t-test, whereas categorical variables were expressed as frequency and percentage (N (%)) and analyzed using the chi-square test or Fisher's exact test, as appropriate. A p-value <0.05 was considered statistically significant.

## Results

A total of 140 full-term pregnant women undergoing primary LSCS were randomized equally into the TXA group (n = 70) and the misoprostol group (n = 70). The two groups were comparable with respect to baseline characteristics, including age, gravidity, and type of LSCS, with no statistically significant differences (all p > 0.05) (Table 1).

**Table 1:** Baseline Characteristics of the Study Participants Data are presented as mean ± standard deviation (SD) for continuous variables and n (%) for categorical variables. Continuous variables were compared using the independent samples t-test, while categorical variables were compared using the chi-square (χ^2^) test. A p-value <0.05 was considered statistically significant. LSCS: lower segment caesarean section; χ^2^: chi-square statistic; df: degrees of freedom

Variable	Category	Tranexamic Acid (n = 70)	Misoprostol (n = 70)	Statistical Value	P-value
Age (years)	Mean ± SD	27.7 ± 6.1	28.0 ± 5.7	t = 0.30	0.764
Gravidity	Primigravida	68 (97.1%)	66 (94.3%)	χ^2^ = 0.70 (df = 1)	0.404
	Multigravida	2 (2.9%)	4 (5.7%)		
Type of LSCS	Elective LSCS	11 (15.7%)	17 (24.3%)	χ^2^ = 1.61 (df = 1)	0.205
	Emergency LSCS	59 (84.3%)	53 (75.7%)		

Women receiving misoprostol had significantly lower mean intraoperative blood loss than those receiving TXA (316.0 ± 158.0 mL vs. 518.5 ± 233.1 mL, p < 0.001). A significantly greater proportion of women in the misoprostol group experienced blood loss of <250 mL (29 (41.4%) vs. 4 (5.7%)), whereas blood loss of >500 mL was more common in the TXA group (29 (41.4%) vs. 11 (15.7%)) (p < 0.001). Women in the misoprostol group also had a significantly smaller postoperative decline in haemoglobin, with 65 (92.9%) women experiencing a fall of <1 g/dL compared with 42 (60.0%) in the TXA group (p < 0.001). The requirement for additional uterotonic agents was significantly lower in the misoprostol group than in the TXA group (2 (2.9%) vs. 11 (15.7%), p = 0.009) (Table 2).

**Table 2:** Comparison of Clinical Outcomes Between the Two Groups Data are presented as mean ± standard deviation (SD) for continuous variables and n (%) for categorical variables. Continuous variables were compared using the independent samples t-test, whereas categorical variables were analysed using the chi-square (χ^2^) test. A p-value <0.05 was considered statistically significant. BP: blood pressure; χ^2^: chi-square statistic; df: degrees of freedom

Variable	Category	Tranexamic Acid (n = 70)	Misoprostol (n = 70)	Statistical Value	P-value
Blood loss (mL)	Mean ± SD	518.5 ± 233.1	316.0 ± 158.0	t = 6.06	<0.001
Blood loss category	<250 mL	4 (5.7%)	29 (41.4%)	χ^2^ = 25.64 (df = 2)	<0.001
	250-500 mL	37 (52.9%)	30 (42.9%)		
	>500 mL	29 (41.4%)	11 (15.7%)		
Fall in haemoglobin	<1 g/dL	42 (60.0%)	65 (92.9%)	χ^2^ = 20.97 (df = 2)	<0.001
	1-2 g/dL	20 (28.6%)	4 (5.7%)		
	>2 g/dL	8 (11.4%)	1 (1.4%)		
Systolic BP (mmHg)	Preoperative	115.0 ± 5.2	116.0 ± 6.8	t = 1.14	0.181
	Postoperative	103.7 ± 7.0	108.7 ± 5.4	t = 4.75	<0.001
Diastolic BP (mmHg)	Preoperative	78.7 ± 4.4	77.2 ± 5.8	t = 1.75	0.082
	Postoperative	69.7 ± 6.1	72.0 ± 6.1	t = 2.23	0.027
Pulse rate (beats/min)	Preoperative	82.1 ± 3.5	82.4 ± 3.6	t = 0.50	0.618
	Postoperative	87.1 ± 4.5	85.3 ± 3.7	t = 2.58	0.011
Additional uterotonic agents required	Yes	11 (15.7%)	2 (2.9%)	χ^2^ = 6.84 (df = 1)	0.009
	No	59 (84.3%)	68 (97.1%)		

The overall incidence and pattern of adverse effects differed between the treatment groups. Fever and shivering occurred significantly more frequently in the misoprostol group, whereas hypotension was significantly more frequent in the TXA group (all p < 0.001). The frequencies of diarrhoea, nausea, and vomiting were comparable between the two groups (all p > 0.05). Blood transfusion was required significantly more frequently in the TXA group than in the misoprostol group (9 (12.9%) vs. 1 (1.4%), p = 0.016). Hysterectomy was required in 2 (2.9%) women in the TXA group and none in the misoprostol group, with no statistically significant difference (p = 0.495) (Table 3).

**Table 3:** Comparison of Adverse Effects and Additional Maternal Interventions Between the Two Groups Data are presented as n (%). Categorical variables were compared using the chi-square (χ^2^) test or Fisher's exact test, as appropriate based on expected cell frequencies. A p-value <0.05 was considered statistically significant. χ^2^: chi-square statistic; df: degrees of freedom

Adverse Effect	Tranexamic Acid (n = 70)	Misoprostol (n = 70)	Statistical Value	P-value
Diarrhoea	9 (12.9%)	8 (11.4%)	χ^2^ = 0.07 (df = 1)	0.792
Nausea	9 (12.9%)	7 (10.0%)	χ^2^ = 0.30 (df = 1)	0.585
Vomiting	9 (12.9%)	4 (5.7%)	χ^2^ = 2.17 (df = 1)	0.140
Hypotension	24 (34.3%)	0 (0.0%)	Fisher's exact	<0.001
Hysterectomy	2 (2.9%)	0 (0.0%)	Fisher's exact	0.495
Blood transfusion required	9 (12.9%)	1 (1.4%)	Fisher's exact	0.016
Fever	4 (5.7%)	19 (27.1%)	χ^2^ = 12.14 (df = 1)	<0.001
Shivering	4 (5.7%)	31 (44.3%)	χ^2^ = 28.06 (df = 1)	<0.001

## Discussion

The present randomized controlled trial compared the efficacy and safety of 600 µg sublingual misoprostol and 1 g intravenous TXA for reducing intraoperative blood loss in women undergoing primary LSCS. To minimize the influence of confounding factors associated with increased haemorrhage, only women with singleton term pregnancies undergoing primary LSCS were included, while those with previous caesarean section, multiple gestation, polyhydramnios, antepartum haemorrhage, induced or augmented labour, obesity, and chorioamnionitis were excluded. Randomization resulted in comparable baseline characteristics between the two groups with respect to age, gravidity, and type of LSCS. Similar inclusion criteria have been adopted in previous randomized trials by Pakniat et al. and Bose and Beegum, facilitating meaningful comparison of the present findings with published literature [19,20].

The principal finding of the present study was that women receiving sublingual misoprostol experienced significantly lower intraoperative blood loss than those receiving intravenous TXA (316.0 ± 158.0 mL vs. 518.5 ± 233.1 mL; p < 0.001). These findings are consistent with those reported by Pakniat et al., who demonstrated significantly lower blood loss in the misoprostol group compared with the TXA group (193.94 ± 104.79 mL vs. 260.25 ± 79.06 mL; p < 0.001) [19]. Although the absolute blood loss reported in their study was lower than that observed in the present study, this difference may be attributed to variations in blood loss estimation techniques. In the present study, blood loss was measured using a standardized gravimetric method by weighing surgical drapes, mops, gauze pieces, and pads before and after surgery in addition to measuring suction bottle contents and blood clots, thereby providing a standardized quantitative assessment of blood loss. In contrast, Bose and Beegum reported lower blood loss with TXA than with misoprostol among women at low risk for PPH, whereas no significant difference was observed among high-risk women [20]. The discrepancy between these findings and those of the present study may reflect differences in study populations, drug dosages, perioperative management protocols, and methods of blood loss estimation.

The postoperative decline in haemoglobin concentration was also significantly lower in the misoprostol group, with a greater proportion of women experiencing a haemoglobin decline of <1 g/dL. This observation is in agreement with the findings of Pakniat et al., who reported a smaller reduction in haemoglobin among women receiving misoprostol compared with TXA [19]. Similarly, Bose and Beegum observed postoperative haemoglobin changes comparable to those reported in the present study [20]. Unlike several earlier studies that assessed postoperative haemoglobin within 24-48 hours, haemoglobin in the present study was measured 72 hours after surgery, providing a standardized postoperative time point for comparison between the treatment groups. However, postoperative haemoglobin decline may be influenced by factors other than intraoperative blood loss, including perioperative fluid administration and physiological haemodynamic changes, and should therefore not be interpreted as a direct measure of blood loss. Furthermore, fewer women in the misoprostol group required additional uterotonic agents or blood transfusion, although these findings should be interpreted in the context of the relatively small number of events. Similar trends have been reported by Pakniat et al., whereas Bose and Beegum found no significant difference in the need for additional uterotonics between treatment groups [19,20].

Both study drugs were generally well tolerated, although their adverse effect profiles differed. Hypotension was observed significantly more frequently in the TXA group, whereas fever and shivering occurred significantly more often among women receiving misoprostol. These findings are consistent with the known pharmacological profile of misoprostol, in which transient pyrexia and shivering are well-recognized dose-related adverse effects. Previous studies by Pakniat et al. and Bose and Beegum also reported nausea, vomiting, and shivering as the most common adverse effects, without any serious maternal complications attributable to either intervention [19,20]. Meta-analyses evaluating TXA during caesarean section have consistently demonstrated that TXA significantly reduces blood loss compared with placebo without increasing major adverse events [21]. However, direct head-to-head comparisons between TXA and misoprostol remain limited. The present findings suggest that, within the study population and perioperative protocol used, sublingual misoprostol was associated with lower measured intraoperative blood loss than intravenous TXA, while the two interventions demonstrated different adverse-effect profiles. These findings should be interpreted in the context of the study's single-centre design and restricted eligibility criteria.

Strengths and limitations

A major strength of the present study is its randomized controlled design, which helps reduce selection bias and improves comparability between the treatment groups. The use of a standardized gravimetric method for estimating intraoperative blood loss, incorporating both the weight of surgical materials and the volume collected in the suction bottle after delivery, provided a more objective quantitative assessment than visual estimation alone. In addition, restricting the study to women with singleton term pregnancies undergoing primary LSCS reduced clinical heterogeneity and enhanced the internal validity of the findings. The study also evaluated both efficacy and safety outcomes, including postoperative haemoglobin changes, adverse drug reactions, and the requirement for additional uterotonics and blood transfusion, thereby providing a broad assessment of the clinical outcomes associated with the two interventions.

However, the study has certain limitations. Being a single-centre study, the findings may not be fully generalizable to other healthcare settings or populations. Owing to the different routes of administration of the study drugs, blinding of participants and treating clinicians was not feasible, introducing the possibility of performance bias, although the primary outcome was assessed using an objective quantitative method. The study included only women undergoing primary LSCS who were at relatively low risk of PPH; therefore, the results cannot be extrapolated to women with repeat caesarean sections or those at high risk for PPH. The relatively small sample size also limits the precision of estimates for less frequent outcomes such as blood transfusion and hysterectomy. Furthermore, long-term maternal outcomes beyond the immediate postoperative period were not evaluated. The use of postoperative haemoglobin decline as a secondary outcome may also be influenced by perioperative fluid administration and other physiological factors. Future multicentre randomized trials with larger sample sizes, inclusion of broader obstetric populations, and longer follow-up are warranted to validate these findings.

## Conclusions

Sublingual misoprostol was associated with lower intraoperative blood loss than intravenous TXA during primary LSCS in women with singleton term pregnancies. Misoprostol was associated with significantly lower blood loss, a smaller postoperative decline in haemoglobin concentration, and a reduced requirement for blood transfusion and additional uterotonic agents, although it was associated with a higher incidence of transient fever and shivering. Both interventions were generally safe and well tolerated. These findings suggest that sublingual misoprostol may be an effective, inexpensive, and easily administered option for reducing intraoperative blood loss during primary LSCS, particularly in resource-limited settings. However, the findings should be interpreted in the context of the single-centre design, relatively small sample size, and restricted study population, and should be confirmed in larger multicentre randomized controlled trials.
